# Evaluation of Online Written Medication Educational Resources for People Living With Heart Failure

**DOI:** 10.1016/j.cjco.2022.07.004

**Published:** 2022-07-12

**Authors:** Simroop Ladhar, Sheri L. Koshman, Felicia Yang, Ricky Turgeon

**Affiliations:** a Faculty of Pharmaceutical Sciences, University of British Columbia, Vancouver, British Columbia, Canada; b Division of Cardiology, Department of Medicine, University of Alberta, Edmonton, Alberta, Canada; c Faculty of Pharmacy and Pharmaceutical Sciences, University of Alberta, Edmonton, Alberta, Canada

## Abstract

**Background:**

Patient educational resources on heart failure (HF) medications may improve patient understanding, which is critical for informed decision-making and patient self-efficacy. The purpose of our study was to evaluate the quality and readability of written medication educational resources available online.

**Methods:**

Two investigators searched Google, Yahoo, and Bing for written patient educational resources that addressed at least one HF medication. We assessed educational quality using the Ensuring Quality Information for Patients (EQIP) tool (range 0 [worst] to 100 [best]), and we evaluated readability using the Flesch-Kincaid Grade Level.

**Results:**

From 693 identified webpages, 39 HF medication educational resources met study eligibility. Among included resources, the median Ensuring Quality Information for Patients score was 61% (interquartile range 54%-68%), with 2 (5%) rated as high quality (score ≥ 75%). The median Flesch-Kincaid Grade Level was 8 (interquartile range 8-12), with 4 (10%) resources meeting the recommended 6th-grade reading level.

**Conclusions:**

Most HF medication educational resources available on the Internet are of acceptable educational quality, but could readily be improved. Most resources were beyond the recommended reading grade level for educational resources, limiting their utility for patients with a low literacy level.

## Graphical abstract

**Figure:**
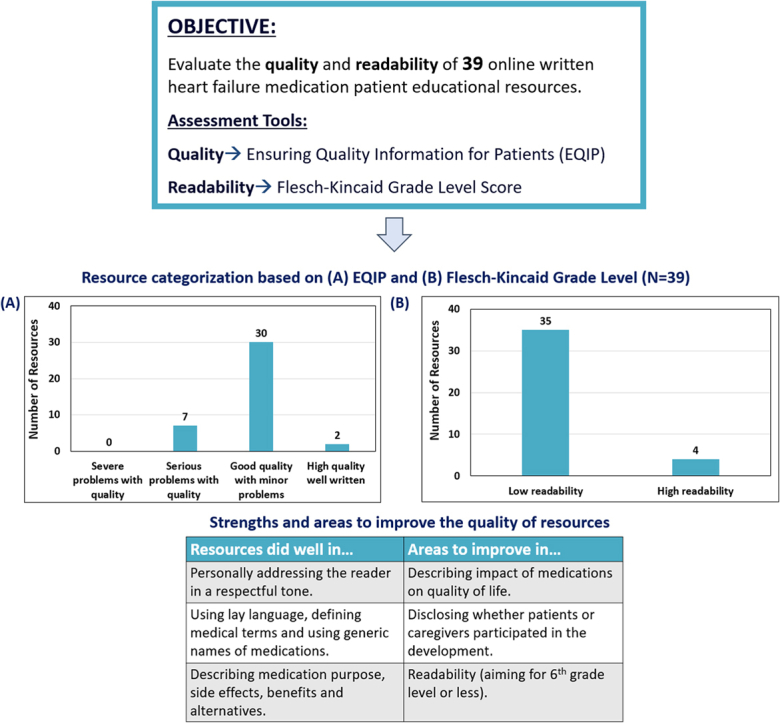

## Résumé

**Contexte:**

Les ressources éducatives destinées aux patients au sujet des médicaments contre l’insuffisance cardiaque (IC) pourraient améliorer la compréhension des patients, ce qui est essentiel pour la prise de décisions éclairées et pour le sentiment d’autoefficacité des patients. L’objectif de notre étude était d’évaluer la qualité et la lisibilité des ressources médicales éducatives écrites en ligne.

**Méthodologie:**

Deux membres de l’équipe de recherche ont utilisé Google, Yahoo et Bing pour repérer les ressources éducatives écrites destinées aux patients et portant sur au moins un médicament contre l’IC. La qualité éducative des ressources a été évaluée avec l’outil « Ensuring Quality Information for Patients » (EQIP), qui fournit avec un score allant de 0 (pire) à 100 (meilleur), et la lisibilité a été évaluée avec le test « Flesch-Kincaid Grade Level ».

**Résultats:**

Sur 693 pages Web repérées, 39 ressources éducatives sur les médicaments contre l’IC répondaient aux critères de notre étude. Pour les ressources évaluées, le score médian sur l’échelle EQIP était de 61 % (intervalle interquartile de 54 % à 68 %), et deux d’entre elles (5 %) ont obtenu un score indiquant une qualité élevée (score ≥ 75 %). Le résultat médian au test Flesch-Kincaid Grade Level était de 8 années de scolarité (intervalle interquartile de 8 à 12), et quatre ressources (10 %) respectaient le niveau de lecture recommandé, qui correspond à une 6^e^ année de scolarité.

**Conclusions:**

La plupart des ressources éducatives en ligne sur les médicaments contre l’IC sont d’une qualité éducative acceptable, mais des améliorations sont possibles. La plupart des ressources corres-pondent à un niveau de lecture plus élevé que ce qui est recommandé pour la rédaction de ressources éducatives, ce qui limite leur utilité pour les patients qui ont un faible niveau de littératie.

Heart failure (HF) affects approximately 750,000 Canadians.^1^ Despite the high morbidity and mortality rates associated with HF, it can be successfully managed with a combination of healthy behaviours, medical devices, and medications.^2^ The variety and complexity of HF treatment options may result in patients and their caregivers feeling overwhelmed, which can negatively impact patient outcomes.^3^^,^^4^ One intervention to overcome this issue is patient education about HF, their disease condition, and available treatment options.^4^^,^^5^ Provision of written patient educational resources may help improve patient understanding of their condition and treatment options.^6^ Various types of educational resources exist, including those designed to enhance patient knowledge (eg, informational pamphlets, patient handouts) and specifically to support shared decision-making (ie, decision aids). Regardless of the type, ideally, written educational resources regarding HF should facilitate discussions between patients and their healthcare providers, should be based on high-quality evidence, and should be developed with the patient’s informational needs and health literacy in mind.^4^

Emerging evidence documents the suboptimal quality and readability of patient educational resources.^7^, ^8^, ^9^, ^10^, ^11^, ^12^, ^13^, ^14^ For instance, a 2014 review assessed the quality of available patient educational resources on left ventricular assist devices, for patients with advanced HF, using the Flesch-Kincaid Grade Level score, the Fry algorithm, and a modified version of the International Patient Decision Aid Standards.^8^ This study found that most educational resources available online that address left ventricular assist devices are of suboptimal quality.^8^ Despite the fact that pharmacotherapy is the cornerstone of HF management, educational resources on HF medication have not yet undergone the same level of rigorous evaluation. Therefore, the purpose of our study was to collect, categorize, and evaluate the quality and readability of online written patient educational resources regarding HF medication.

## Methods

### Search strategy

From May 2021 to June 2021, 2 investigators (S.L. and F.Y.) independently conducted simultaneous, comprehensive searches of the top 3 Internet search engines—Google, Yahoo, and Bing—using Google Chrome (the most commonly used browser) to collect Internet-based educational resources about HF medications.^7^^,^^9^^,^^11^^,^^15^^,^^16^ Search queries included the following: (i) “Heart failure medication patient information”; (ii) “Heart failure medications patient information”; (iii) “Heart failure medication patient handout”; (iv) “Heart failure medications patient handout”; (v) “Heart failure drugs patient information”; and (vi) “Heart failure drugs patient handout”. The search queries used were developed through discussion and collaboration between the researchers, with the goal of replicating search terms that patients looking for this information might use (ie, more general, broad terms, in lay language). The investigators used an empirical method to minimize the influence of prior search history, which involved clearing the cookies from their web browsers and entering “incognito mode” prior to each search. To emulate typical Internet searches of patients looking for health information, we considered the results from the first 2 pages of each search.^11^^,^^17^, ^18^, ^19^ The number of results per page was 10 for Google and Yahoo, and 8-10 for Bing, depending on ads. Additionally, we manually searched Web sites from key cardiovascular, HF, pharmacy, and patient advocacy organizations; HF medication manufacturers; and the Ottawa Decision Aid Inventory (Supplemental Table S1). Key Web sites were defined as being those that are well known Web sites of cardiovascular and HF societies, prominent Canadian cardiac centres, and health authorities. Evaluators assessed resources as a whole and multiple pages, if relevant, but hyperlinks to different URLs were not assessed. Further, S.L. and F.Y. individually removed any intra-assessor duplicate resources from their results prior to combining their lists. After the results were combined, between-assessor duplicates were removed. Lastly, although we were unable to determine the exact intention of the resources evaluated, the inferred intention was that they were developed to provide patient education. Therefore, we included resources irrespective of their stated (or unstated) intention if they met all the eligibility criteria below.

### Eligibility criteria

We included educational resources that met the following criteria: (1) written resources directed at patients; (2) written in English; (3) included ≥ 10 sentences about HF medications (as per Iacovetto et al. 2014^8^) to ensure inclusion of resources with substantive content on medications; (4) accessible without having to register and/or pay a fee; (5) specifically described ≥ 1 different HF medication treatment option (i.e. individual medication or drug class); and (6) provided medication information specific to their use in HF.

### Primary outcome: EQUIP score

The primary outcome was educational resource quality, based on the Ensuring Quality Information for Patients (EQIP) score. EQIP is a validated 20-item questionnaire first described in 2004 that measures the quality of patient information on a scale from 0% (worst) to 100% (best) by assessing the domains of completeness, appearance, understandability, and usefulness (Supplemental Appendix S1).^5^ Each question has 3 possible answers with an accompanying score of 1 (yes), 0.5 (partly), or 0 (no). The total EQIP score was determined by averaging individual items’ scores using the following formula: [((# of Yes∗1) + (# of Partly∗0.5) + (# of No∗0))/20 – (# of does not apply))] ∗100 = % score.^5^ Domains that were not applicable to a given resource were excluded from the overall score calculation. Two reviewers (F.Y. and S.L.) evaluated each resource using the EQIP score, with a discrepancy between reviewers defined as a difference of ≥ 10% in EQIP score. Discrepant scores, which could occur due to the subjective nature of some of the questions, were reviewed by the 2 reviewers in order to reach a consensus, with disagreements resolved by a third author (R.T.). Following resolution of discrepant scores, the 2 EQIP scores calculated for each resource were averaged. This final averaged score was used in the analysis and to categorize each resource as one of the following: (i) well written, high-quality (score of 76%-100%); (ii) good quality with minor problems (score of 51%-75%); (iii) serious problems with quality (score of 26%-50%); and (iv) severe problems with quality (score of 0%-25%), consistent with categories recommended in the original EQIP development publication.^5^

### Secondary outcome: readability

Readability was assessed primarily using the Flesch-Kincaid Grade Level score, and secondarily, via the Flesch Reading Ease score, by copying the text of each resource into a Microsoft Word 2016 (Microsoft, Redmond, WA) document and using the software’s built-in readability function. The Flesch-Kincaid Grade Level and the Flesch Reading Ease scores have previously been validated and used to measure the readability of patient educational resources.^8^^,^^12^^,^^13^ In the US, an estimated 61% of adults read at a 6th-grade level,^9^ and it is therefore recommended that patient information resources be written at a 6th- grade or lower reading level.^12^^,^^13^^,^^20^ For this study, HF medication patient educational resources with a higher than 6th^-^grade reading level were considered to have low readability. We further used a Flesch Reading Ease score < 80 to indicate low readability.^13^^,^^21^^,^^22^ Lastly, we collected data on each resource’s total word count, for descriptive purposes.

### Statistical analysis

We performed descriptive analyses, and we report data using medians and interquartile ranges (IQRs) for continuous variables, and frequencies and percentages for categorical variables. All analyses were performed using Microsoft Excel 2016 (Microsoft, Redmond, WA).

## Results

**Figure 1:**
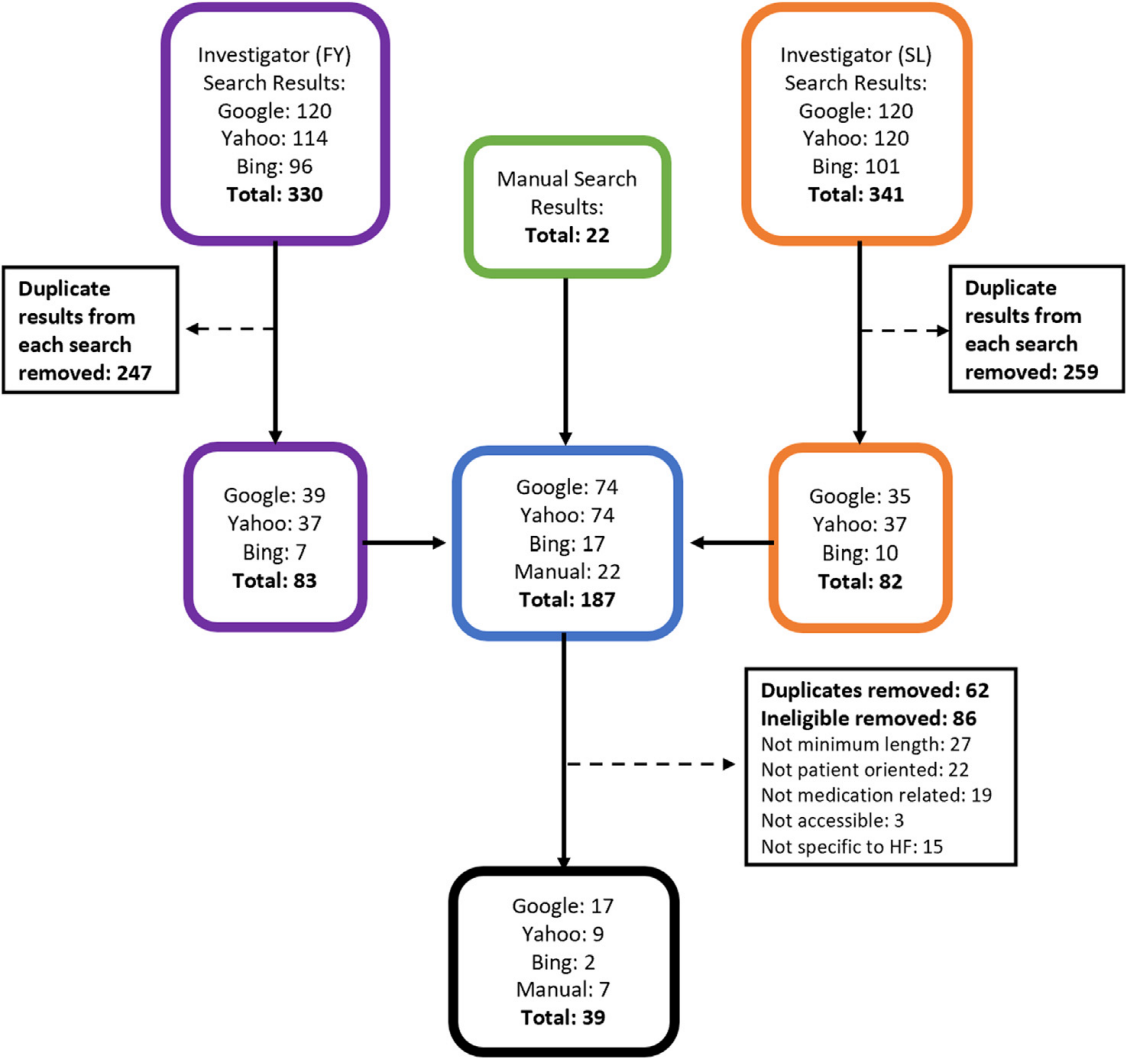
Study flow diagram. HF, heart failure.

From 693 search results, we identified 39 HF medication educational resources that met our eligibility criteria (Fig. 1). Despite the methods employed and the fact that the searches conducted were identical, F.Y.’s and S.L.’s search results differed slightly, likely due to differences in geographic location (Alberta vs British Columbia). Key characteristics of HF medication educational resources are described individually in Table 1, and summary statistics are provided in Table 2.

**Table 1:** Individual characteristics and quality assessment of 39 HF medication educational resources, in descending order of EQIP score

Resource title	Developer/ funding source	EQIP Score, %	Flesch-Kincaid Grade Level	Word count
A Marvellous Guide to Medicines for Heart Failure https://pumpingmarvellous.org/wp-content/uploads/2018/05/Heart-Failure-Medicine-Guide.pdf	Pumping Marvellous	79	8.6	9039
Patient Education: Heart Failure (Beyond the Basics) https://www.uptodate.com/contents/heart-failure-beyond-the-basics	UpToDate	76	9	4778
Heart Failure - A Guide for Patients and Families https://www.ottawaheart.ca/sites/default/files/uploads/heart-failure-patient-guide.pdf	University of Ottawa Heart Institute	73	8	7571
Heart Failure http://www.cardiacbc.ca/health-info/heart-conditions/heart-failure	Cardiac Services British Columbia	72	8	1020
Living With Heart Failure https://www.heartandstroke.ca/-/media/pdf-files/canada/health-information-catalogue/en-living-with-heart-failure.pdf	Heart & Stroke Foundation	71	6	20,853
Heart Failure Patient Education Handbook https://www.asante.org/app/files/public/2134/Heart-Failure-Handbook.pdf	Asante	71	7.5	4717
Heart Failure Diagnosis & Treatment https://www.mayoclinic.org/diseases-conditions/heart-failure/diagnosis-treatment/drc-20373148	Mayo Clinic	71	9	4518
Digoxin: A Medicine for Heart Problems https://familydoctor.org/digoxin-a-medicine-for-heart-problems/?adfree=truetreat-heart-failure	familydoctor.org	70	6.5	929
Heart Failure Medications https://www.ottawaheart.ca/heart-failure-patient-guide/heart-failure-medications	University of Ottawa Heart Institute	69	10	1638
Medications to Manage Heart Failure https://www.health.qld.gov.au/__data/assets/pdf_file/0030/993009/Medications-booklet-web-version.pdf	Queensland Government	69	10	3068
A Decision Aid for Entresto https://www.cardiosmart.org/docs/default-source/assets/decision-aid/heart-failure-drug-options.pdf?sfvrsn=aaff9c98_1	American College of Cardiology (CardioSmart)	69	8.7	1024
Introduction to Medications https://ourhearthub.ca/medications/	Ted Rogers Institute for Heart Research	67	8.1	759
Heart Failure https://myhealth.alberta.ca/Health/pages/conditions.aspx?hwid=hw44415#tp17546	Alberta Health Services	67	6.2	6062
Congestive Heart Failure https://patient.info/heart-health/heart-failure-leaflet	patient.Info	65	8.7	2032
What Medications Are Used to Treat Heart Failure? https://heart-failure.net/medications	heartfailure.net	65	11.3	1077
A Quick Guide to Living With Heart Failure https://www.novartis.com/sites/novartis_com/files/nvs-hf-patient-booklet.pdf	Novartis / Pumping Marvellous	64	8	4070
Heart Failure Medications https://my.clevelandclinic.org/departments/heart/patient-education/recovery-care/heart-failure/medications	Cleveland Clinic	64	6.4	2059
Managing Heart Failure https://www.coeuretavc.ca/-/media/pdf-files/canada/health-information-catalogue/en-managing-heart-failure-v3.ashx	Heart & Stroke Foundation	62	7.3	7400
What to Expect: Living With Heart Failure https://www.upmc.com/-/media/upmc/patients-visitors/education/documents/living-with-heart-failure-booklet.pdf	University of Pittsburgh Medical Center	61	8	5458
Commonly Prescribed Heart Failure Medications https://heartfailureoxford.org.uk/patient/heart-failure-medications/	Heart Failure Oxfordshire	61	9.5	1531
Discharge Packet for Patients Diagnosed With Heart Failure https://www.heart.org/-/media/files/health-topics/heart-failure/hf-discharge-packet.pdf?la=en&hash=90463681A07EE6230276BC27A08F5D337D1D6D8C	American Heart Association	60	6	11,630
Medicines for Congestive Heart Failure https://healthy.kaiserpermanente.org/washington/health-wellness?item=/common/healthAndWellness/conditions/heartDisease/chfMedications.html	Kaiser Permanente	58	9.2	1045
Everything You Need to Know About Heart Failure Medications https://www.healthline.com/health/heart-failure/heart-failure-medications	Healthline	58	10	1983
About Heart Failure https://www.cardiomyopathy.org/about-cardiomyopathy/heart-failure	Cardiomyopathy.org	58	10.2	890
CHF Booklet https://www.meritushealth.com/documents/CHF-booklet.pdf	Meritus Health	56	9	5230
Heart Failure https://www.farxiga.com/heart-failure	AstraZeneca	56	10	3081
HF Patient Education Booklet http://www.gov.pe.ca/photos/original/hpei_cp_hf_book.pdf	Prince Edward Island Government	55	6.7	4078
Heart Failure Handbook https://www.thechristhospital.com/Documents/Our%20Services/Heart%20Failure%20Handbook.pdf	The Christ Hospital	55	7	2120
Heart Failure—Treatment https://www.nhs.uk/conditions/heart-failure/treatment/	United Kingdom National Health Services	54	11	1933
10 Drugs Commonly Prescribed for Heart Failure https://www.healthgrades.com/right-care/heart-failure/10-drugs-commonly-prescribed-for-heart-failure	HealthGrades	54	8	1428
Self-Care Guide for the Heart Failure Patient^30^	American Heart Association	54	9.6	1020
Heart Failure Medicines https://www.heartfailurematters.org/what-your-doctor-can-do/heart-failure-medicines/	heartfailuremattters.org	53	10	437
Treatment of Heart Failure? https://www.aahfn.org/mpage/treatement_hf	American Association of Heart Failure Nurses	50	7.5	2089
Understanding Heart Failure: Answers to Common Questions https://www.med.umich.edu/1libr/CCG/HeartFailure.pdf	Michigan Medicine	49	7.5	1328
Congestive Heart Failure Medications https://www.medicinenet.com/congestive_heart_failure_medications/drug-class.htm	MedicineNet	49	12	1328
Heart Failure https://www.nhlbi.nih.gov/health/heart-failure	National Heart Lung and Blood Institute	49	8	5269
Heart Failure Medicines https://myhealth.alberta.ca/Alberta/AlbertaDocuments/Heart-Failure-Medicines-Oct-2019.pdf	Alberta Health Services	46	7.3	886
Medications https://www.cardiosmart.org/topics/heart-failure/treatment/medications	American College of Cardiology (CardioSmart)	38	8.4	313
Heart Failure Treatments https://www.ucsfhealth.org/conditions/heart-failure/treatment	University of California San Francisco Health	31	11	1272

EQIP scores range from 0% (worst) to 100% (best).

CHF, congestive heart failure; EQUIP, Ensuring Quality Information for Patients; HF, heart failure.

**Table 2:** Summary characteristics and quality assessment of heart failure medication educational resources

Characteristic	Value	First and third quartile (range)
Word count	2032	1045 to 4778 (313 to 20,853)
Format		
Webpage	25 (64)	**—**
PDF	14 (36)	**—**
Country of origin		
USA	22 (56)	**—**
United Kingdom	7 (18)	**—**
Canada	9 (23)	**—**
Australia	1 (3)	**—**
EQIP, median	61	54 to 69 (31 to 79)
High quality	2 (5)	—
Good quality, minor problems	30 (77)	—
Serious problems	7 (18)	—
Severe problems	0	—
Readability		
Flesch-Kincaid Grade Level, median	8	8 to 10 (6 to 12)
≤6th grade	4 (10)	—
Flesch Reading Ease, median	59	52 to 65 (36 to 72)
≥ 80	0	—

Values are n (%), unless otherwise indicated.

EQIP, Ensuring Quality Information for Patients score; PDF, portable document format.

### EQIP

**Figure 2:**
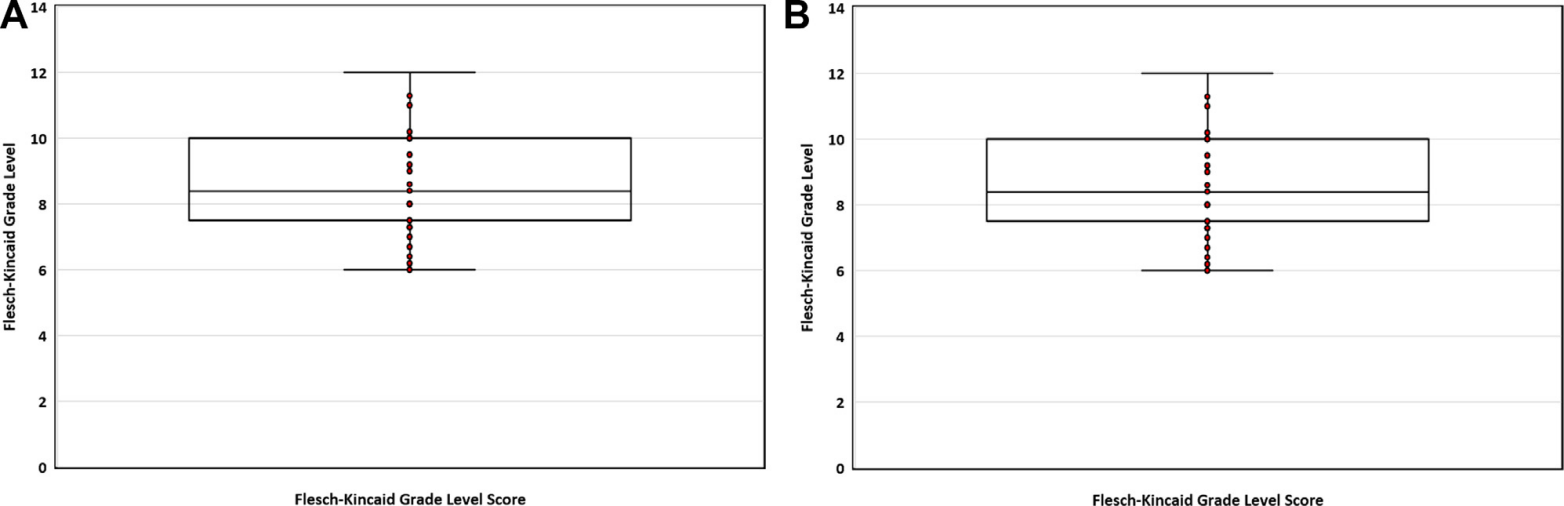
Boxplot of (**A**) Ensuring Quality Information for Patients (EQIP) score and (**B**) Flesch-Kincaid Grade Level for 39 heart failure medication educational resources.

The median EQIP score was 61% (IQR 54% to 68%; Table 2; Fig. 2A). Of the 39 included educational resources, 2 (5%) were of high quality, 30 (77%) were of good quality with minor problems, and 7 (18%) had serious problems with quality. Assessment of the specific components of the total EQIP score revealed that 36 resources (92%) used lay terms, 38 (97%) addressed the reader personally, and 39 (100%) had a respectful tone. However, only 2 resources (5%; including the highest-scoring resource) reported consulting with patients/family members during development. In terms of medication-specific information, 38 resources (97%) addressed the purpose of described HF medications, 28 (72%) qualitatively described benefits (eg, decrease mortality), 28 (72%) qualitatively described the medication risks and side-effects, and 21 (54%) addressed alternative medications (eg, angiotensin-receptor blocker if cough develops while taking an angiotensin-converting enzyme inhibitor). Further, 34 resources (87%) used generic names (instead of, or in addition to, brand names) and distinguished brand names as such, whereas the remaining 5 resources (13%) only discussed medication classes. Lastly, 24 resources (62%) did not address quality-of-life issues, with the remaining 15 (38%) only partly addressing this item. Only 5 resources (13%) included visual content to supplement the text.

### Readability

The median Flesch-Kincaid Grade Level was an 8^th^-grade reading level (IQR 8th to 10th grade) (Fig. 2B). Only 4 resources (10%) achieved the recommended 6th-grade reading level, and no resource had a 5th-grade or lower reading level. The median Flesch Reading Ease score was 59 (IQR 52 to 65), with all resources demonstrating low readability based on this score.

## Discussion

HF is a chronic, complex condition that can be treated using a variety of healthy behaviours, medical devices, and medications.^2^ Provision of written resource for patients to review on their own time and at their own pace can significantly improve information retention, compared to verbal instruction.^4^^,^^5^ In this study, we found that most online HF medication patient educational resources were of good quality with minor problems, based on the EQIP tool. However, most HF medication educational resources were written at a grade level beyond the reading level of most people in the general population.

We identified several key areas for improvement among HF medication patient educational resources. In general, resources had a respectful tone, used generic names of medications, presented information in a logical order, and qualitatively described the purpose, side-effects, benefits (though not quantitatively,) and alternatives of medications. However, few followed a summative flow (ie, outlining the document content and covering what is stated), involved patients and caregivers in their development, or addressed quality-of-life issues.

Having a clear definition outlining the document content can help patients, caregivers, and healthcare providers understand whether the resource is suitable for the patient’s specific informational needs.^5^^,^^23^^,^^24^ Next, patients and healthcare providers may have different perceptions of what information is important to convey in educational materials.^5^^,^^23^^,^^25^^,^^26^ Therefore, involving patients and caregivers in development of resources can help tailor educational materials to the needs of patients, making them more useful and relevant to their target audience. Further, describing the quality-of-life and practical impacts of medications ensures resource transparency, enables realistic patient expectations,^5^^,^^27^ and allows patients to better understand and implement self-care and management strategies.^5^^,^^24^ A recent study established a framework for patient-oriented practical issues to be included in evidence summaries and shared decision-making tools.^28^ Such practical considerations align closely with quality-of-life issues incorporated into the EQIP questionnaire (eg, how a treatment impacts a patient’s daily routine or social life) and should be included in HF medication educational resources.^5^^,^^28^ These details are also more likely to align with patients’ individual goals of therapy (eg, improve symptoms, daily functioning, capacity for social interaction, etc.) and are crucial to include in patient education to engage patients in their care and medication management.^3^

Our search identified resources with a wide range of readability, with few resources achieving sufficient readability. The readability level of HF medication educational resources thereby poses a problem, as patients may not be able to fully comprehend the included information, regardless of its quality. An additional consideration for barriers to use of educational resources is the length of time required to read through them. The average adult reads at a pace of approximately 250 words/minute.^29^ Of the top 5 highest-scoring resources based on the EQIP tool, the median word count was 4778, indicating approximately 19 minutes of reading time. For the average patient, who may be new to their diagnosis and receiving an overwhelming amount of information, resource length is likely a substantial barrier to absorbing and implementing the included information.^3^

## Limitations

Limitations to this study warrant discussion. First, we restricted resource inclusion to written materials available online. We did not capture information presented within social media communities (eg, Reddit, Facebook, etc.), video resources (eg, YouTube), or local/clinic-specific resources that are not available online. We anticipate greater variability of quality of these resources, as they may be subject to less curation. Second, the EQIP instrument was open to interpretation, which resulted in initial variability in scoring between the 2 reviewers. However, we mitigated this variability by incorporating duplicate scoring and an *a priori* plan to identify and resolve discrepancies. Third, the EQIP questionnaire used for our primary outcome defines quality from the perspective of healthcare professionals, rather than from the perspective of the end-user (ie, the patient). Therefore, it may not encompass all domains of quality or treatment considerations that are important to patients, or capture the relative value that patients place on each domain.

## Conclusions

Most HF medication educational resources available on the Internet are of acceptable educational quality, but could readily be improved. Most resources were beyond the recommended reading grade level for educational resources, limiting their utility for patients with a low literacy level.

## Funding Sources

The authors have no funding sources to declare.

## Disclosures

The authors have no conflicts of interest to disclose.

## References

[1] Government of Canada Canadian Chronic Disease Surveillance System (CCDSS). Available at:. https://health-infobase.canada.ca/ccdss/data-tool/.

[2] McDonald M., Virani S., Chan M. (2021). CCS/CHFS heart failure guidelines update: defining a new pharmacologic standard of care for heart failure with reduced ejection fraction. Can J Cardiol.

[3] McHorney C.A., Mansukhani S.G., Anatchkova M. (2021). The impact of heart failure on patients and caregivers: a qualitative study. PloS One.

[4] Boyde M., Peters R. (2014). Education material for heart failure patients: what works and what does not?. Curr Heart Fail Rep.

[5] Moult B., Franck L.S., Brady H. (2004). Ensuring quality information for patients: development and preliminary validation of a new instrument to improve the quality of written health care information. Health Expect.

[6] van Weel C. (2002). More self reliance in patients and fewer antibiotics: still room for improvement. BMJ.

[7] Nassiri M., Mohamed O., Berzins A. (2016). Surfing behind a boat: quality and reliability of online resources on scaphoid fractures. J Hand Surg Asian Pac Vol.

[8] Iacovetto M.C., Matlock D.D., McIlvennan C.K. (2014). Educational resources for patients considering a left ventricular assist device: a cross-sectional review of Internet, print, and multimedia materials. Circ Cardiovasc Qual Outcomes.

[9] Hameed I., Hameed N.U.F., Oakley C.T. (2021). Systematic assessment of online health information for coronary revascularization. JAMA Intern Med.

[10] Hazelton G., Al-Khatib Sears S. (2013). Assessment of the quality of existing patient educational tools focused on sudden cardiac arrest: a systematic evaluation by the Sudden Cardiac Arrest Thought Leadership Alliance. Patient Prefer Adherence.

[11] Engelmann J., Fischer C., Nkenke E. (2020). Quality assessment of patient information on orthognathic surgery on the Internet. J Craniomaxillofacial Surg.

[12] Dalziel K., Leveridge M.J., Steele S.S., Izard J.P. (2016). An analysis of the readability of patient information materials for common urological conditions. Can Urol Assoc J.

[13] Kher A., Johnson S., Griffith R. (2017). Readability assessment of online patient education material on congestive heart failure. Adv Prev Med.

[14] Lee K.S., Cho Y.M., Oh S.H., Jung M.S., Yoon J.Y. (2021). Evaluation of the heart failure in Internet patient information: descriptive survey study. Int J Environ Res Public Health.

[15] NetMarketShare Market share statistics for Internet technologies. Available at:. http://www.netmarketshare.com.

[16] statcounter Search engine market share worldwide. Available at:. https://gs.statcounter.com/search-engine-market-share.

[17] Eysenbach G., Kohler C. (2002). How do consumers search for and appraise health information on the world wide web? Qualitative study using focus groups, usability tests, and in-depth interviews. BMJ.

[18] Aldairy T., Laverick S., McIntyre G.T. (2012). Orthognathic surgery: Is patient information on the Internet valid?. Eur J Orthod.

[19] Karamitros G.A., Kitsos N.A. (2018). Clefts of the lip and palate: is the Internet a trustworthy source of information for patients?. Int J Oral Maxillofac Surg.

[20] Weiss BD. Removing barriers to better, safer care. Health literacy and patient safety: Help patients understand. In: Manual for Clinicians, 2nd ed. 2007. Available at: http://www.partnershiphp.org/Providers/HealthServices/Documents/Health%20Education/CandLToolKit/2%20Manual%20for%20Clinicians.pdf. Accessed August 26, 2021.

[21] Edmunds M.R., Barry R.J., Denniston A.K. (2013). Readability assessment of online ophthalmic patient information. JAMA Ophthalmol.

[22] Flesch R. (1948). A new readability yardstick. J Appl Psychol.

[23] Coulter A., Entwistle V.A., Gilbert D. (1999). Informing patients: an assessment of the quality of patient information materials. BMJ.

[24] Department of Health (2002). https://wessexahsn.org.uk/img/projects/NHS%20Toolkit%20for%20producing%20patient%20information%20v2%20(2003)-1489154340.pdf.

[25] Perkins L. (2001). Developing a tool for health professionals involved in producing and evaluating nutrition education leaflets. J Human Nutr Diet.

[26] Coulter A. (1998). Evidence based patient information. BMJ.

[27] Morris K.C. (2001). Psychological distress in carers of head injured individuals: the provision of written information. Brain Injury.

[28] Heen A.F., Vandvik P.O., Brandt L. (2021). A framework for practical issues was developed to inform shared decision-making tools and clinical guidelines. J Clin Epidemiol.

[29] Brysbaert M. (2019). How many words do we read per minute? A review and meta-analysis of reading rate. J Mem Lang.

[30] White M.F., Kirschner J., Hamilton M.A. (2014). Self-care guide for the heart failure patient. Circulation.

