# Supplementary material for: Evaluation of Online Written Medication Educational Resources for People Living With Heart Failure
Source: CJC Open. 2022 Jul 12;4(10):858–65. doi: 10.1016/j.cjco.2022.07.004 (PMC9568683; doi:10.1016/j.cjco.2022.07.004)
Supplement: Supplemental Table [file mmc1.docx]

# Supplemental Table S1. Key websites searched manually.

| **Website** |
| --- |
| acc.org |
| albertahealthservices.ca/info/Page7735.aspx |
| bsh.org.uk/resources/heart-failure-information/ |
| cardiacbc.ca/ |
| cardiosmart.org/ |
| ccs.ca/ |
| cdc.gov/heartdisease/resources_for_patients.htm |
| corhealthontario.ca/resources-for-healthcare-planners-&-providers/integrating-heart-failure-care/Heart-Failure-Education |
| escardio.org/The-ESC |
| heart.org |
| heartandstroke.ca/ |
| heartandstroke.ca/heart-disease/conditions/heart-failure |
| heartfailure.ca/ |
| hfsa.org/patient |
| medlineplus.gov/ency/patientinstructions/000364.htm |
| myhealth.alberta.ca/Health/pages/conditions.aspx?hwid=hw44415 |
| ottawaheart.ca/heart-condition/heart-failure |
| rxfiles.ca |
| tedrogersresearch.ca/why-heart-failure/visit-patient-website/ |

# Supplemental Appendix S1. Ensuring Quality Information for Patients (EQIP) Questionnaire

| **Question** | **Rating**  **1=Yes,0.5=Partly,0=No,N/A*** |
| --- | --- |
| 1. Does the document start by telling you what it will cover and then cover what it says? |  |
| 2. Does it use everyday language, explaining unusual or medical words or abbreviations or jargon? |  |
| 3. Does it use short sentences of less than 15 words on average? |  |
| 4. Does it personally address the reader? |  |
| 5. Is the tone respectful? |  |
| 6. Is the design and layout of the document satisfactory? |  |
| *7. Does the document contain easy-to-understand illustrations, diagrams or photos that are relevant to the subjects it covers? |  |
| 8. Is the information presented in logical order? |  |
| *9. Does the document have a named space for reader to make notes? |  |
| 10. Does the document contain contact details for the healthcare services where the reader can receive care or treatment for problems discussed in the document? |  |
| 11. Does the document contain the date it was produced? |  |
| 12. Does the document contain the name of the person or department that produced it? |  |
| 13. Does the document say whether patients and/or their families were involved or consulted in its production? |  |
| *14. Does it use generic names for all medications or products instead of, or in addition to, brand names and does it designate brand names as such? |  |
| 15. Does the document address quality of life issues, like school attendance or reduced mobility? |  |
| 16. Does the document contain details of other sources of information for the reader, such as support organisations or website? |  |
| 17. Is the purpose described? |  |
| 18. Are the benefits described? |  |
| 19. Are risks and side-effects described? |  |
| 20. Are any alternatives described? |  |
|  | |
| **Question** | **Reasoning for N/A*** |
| Does the document contain easy-to-understand illustrations, diagrams or photos that are relevant to the subjects it covers? | The document was clearly intended to be written as an article and therefore does not contain infographics etc. |
| Does the document have a named space for reader to make notes? | For websites, this question was irrelevant as it only applies to resources in a PDF format. |
| Does it use generic names for all medications or products instead of, or in addition to, brand names and does it designate brand names as such? | For educational resources discussing drug classes (as opposed to individual drug names) this irrelevant. |

*Reference: Moult B, Franck LS, Brady H. Ensuring quality information for patients: development and preliminary validation of a new instrument to improve the quality of written health care information. Health Expect. 2004;7(2):165-75. doi: 10.1111/j.1369-7625.2004.00273.x*
